# Chronic creatine kinase deficiency eventually leads to congestive heart failure, but severity is dependent on genetic background, gender and age

**DOI:** 10.1007/s00395-012-0276-2

**Published:** 2012-07-04

**Authors:** Craig A. Lygate, Debra J. Medway, Philip J. Ostrowski, Dunja Aksentijevic, Liam Sebag-Montefiore, Imre Hunyor, Sevasti Zervou, Jurgen E. Schneider, Stefan Neubauer

**Affiliations:** Department of Cardiovascular Medicine, Wellcome Trust Centre for Human Genetics, University of Oxford, Roosevelt Drive, Oxford, OX3 7BN UK

**Keywords:** Creatine kinase, Cardiac energetics, Heart failure, Energy metabolism, Transgenic mice

## Abstract

The creatine kinase (CK) energy transport and buffering system supports cardiac function at times of high demand and is impaired in the failing heart. Mice deficient in muscle- and mitochondrial-CK (M/Mt-CK^−/−^) have previously been described, but exhibit an unexpectedly mild phenotype of compensated left ventricular (LV) hypertrophy. We hypothesised that heart failure would develop with age and performed echocardiography and LV haemodynamics at 1 year. Since all previous studies have utilised mice with a mixed genetic background, we backcrossed for >10 generations on to C57BL/6, and repeated the in vivo investigations. Male M/Mt-CK^−/−^ mice on the mixed genetic background developed congestive heart failure as evidenced by significantly elevated end-diastolic pressure, impaired contractility, LV dilatation, hypertrophy and pulmonary congestion. Female mice were less severely affected, only showing trends for these parameters. After backcrossing, M/Mt-CK^−/−^ mice had LV dysfunction consisting of impaired isovolumetric pressure changes and reduced contractile reserve, but did not develop congestive heart failure. Body weight was lower in knockout mice as a consequence of reduced total body fat. LV weight was not significantly elevated in relation to other internal organs and gene expression of LVH markers was normal, suggesting an absence of hypertrophy. In conclusion, the consequences of CK deficiency are highly dependent on genetic modifiers, gender and age. However, the observation that a primary defect in CK can, under the right conditions, result in heart failure suggests that impaired CK activity in the failing heart could contribute to disease progression.

**Electronic supplementary material:**

The online version of this article (doi:10.1007/s00395-012-0276-2) contains supplementary material, which is available to authorized users.

## Introduction

Creatine kinase (CK) is the major phosphotransfer system in the heart linking energy production to energy utilisation. Mitochondrial-CK (Mt-CK) catalyses the transfer of a phosphoryl group from ATP to creatine to form phosphocreatine (PCr), which transports energy within the cell and acts as a short-term energy buffer, available for rapid regeneration of ATP at times of suddenly increased workload. This reverse reaction is catalysed by cytosolic CK dimers, consisting of muscle- (M-CK) and brain- (B-CK) isoforms, with the MM-CK isoenzyme by far the most prevalent [12, 32].

Down-regulation of the CK system is a hallmark of heart failure regardless of aetiology [29]. For example, total creatine is reduced by up to 56 % [18] and CK activity by up to 48 % [33], and low PCr/ATP ratio is a predictor of mortality in patients with dilated cardiomyopathy [20], contributing to the hypothesis that the failing heart is energy starved [19]. Understanding how such changes impact on pathophysiology may inform new therapeutic approaches, and in support of this, over-expression of M-CK has recently been shown to protect mice from heart failure due to pressure overload [11], suggesting CK changes are not simply an epiphenomenon.

One approach is to study mice with genetic deletion of key components of the CK system and then determine the effect on cardiac phenotype in vivo. At the simplest level, overt dysfunction would indicate potential for a causative role in the progression to heart failure, but in practice, interpretation of knockout models is often obfuscated by physiological redundancy and compensatory adaptations [2]. CK knockout strains have been created for M-CK, Mt-CK and the combined double gene deletion [25, 27], and have proven to be no exception. Loss of M-CK (~50 % of total CK activity) results in a very mild phenotype with no overt left ventricular hypertrophy (LVH) or dysfunction apparent [16, 28, 30]. The double KO (M/Mt-CK^−/−^) has a more robust cardiac phenotype, which is broadly similar to the single Mt-CK^−/−^, suggesting that loss of Mt-CK rather than up to 98 % loss of total creatine kinase activity is the driving force [16, 22, 24]. Compensated LVH has been observed in most studies [13, 16, 17] (although not in all [22, 24]), and likewise functional defects have not always been evident [22] or relate only to impaired contractile reserve [7]. Even at 41 weeks of age, there were no differences in MRI parameters of global function [16], but only subtle changes in perfusion and ejection times [17]; remarkably, these mice could survive experimental myocardial infarction [16].

However, a number of question marks remain concerning the in vivo cardiac phenotype of the M/Mt-CK^−/−^ mice. Firstly, it is not known whether LVH will eventually progress to heart failure if studied beyond 41 weeks of age. Secondly, LV haemodynamic measurements have never been obtained in vivo, but only at much lower workloads in Langendorff preparations. Thirdly, all studies to date have used mice on a mixed C57BL/6 and 129Sv genetic background. We have previously shown this to be critically important in Mt-CK^−/−^ mice, where backcrossing to a pure BL/6 background resulted in loss of the LVH phenotype [15]. In the current study we address all of these points. We demonstrate for the first time that M/Mt-CK^−/−^ mice on the standard mixed background eventually develop congestive heart failure by 1 year of age. We then characterise a backcrossed strain of M/Mt-CK^−/−^ on a pure BL/6 background and show this to have defects in isovolumetric contraction/relaxation and reduced body fat, but to no longer develop LVH or congestive heart failure. Our findings show for the first time that a chronic primary defect in creatine kinase ultimately results in congestive heart failure, but that severity of dysfunction is influenced by sex and genetic modifiers.

## Methods

### Mouse colonies

**Fig. 1:**
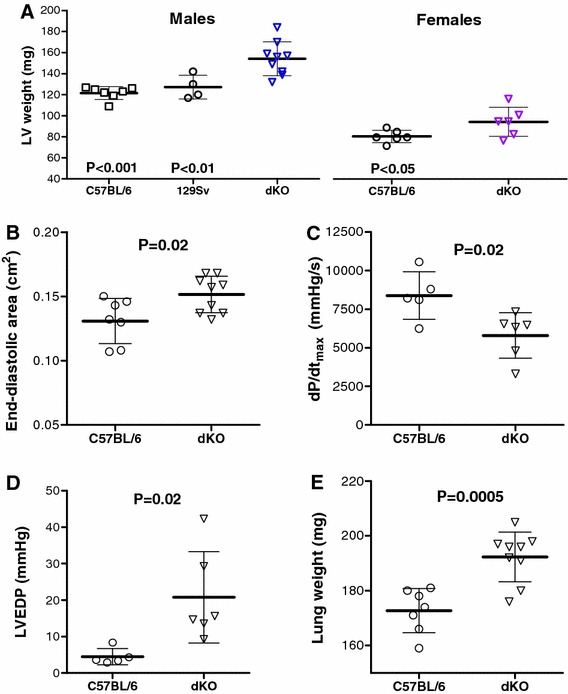
At 1-year of age, M/Mt-CK^−/−(129/Bl6)^ mice on a mixed genetic background (dKO) develop left ventricular (LV) hypertrophy and dysfunction. LV hypertrophy is significant regardless of whether C57BL/6 or 129SV is used as control strain, and occurs in both males and females (*Panel* ***a***). *Panels* ***b*** *–* ***e*** are from males only (*n* = 5–7, C57BL/6; *n* = 6–9, dKO) and indicate significant LV dilatation in KO mice measured by echocardiography (**b**); impaired LV contractility (**c**) and elevated end-diastolic pressure (**d**) by in vivo haemodynamics; and pulmonary congestion in dKO mice at post-mortem (**e**). Values are mean ± SD

Creatine kinase double knockout mice (i.e. deficient in both M-CK and mitochondrial-CK isoenzymes) were imported from a colony at the University of Würzburg, which in turn originated from the laboratory of Prof Wieringa (University of Nijmegen, The Netherlands) [25, 27]. These represent the standard strain reported in the literature, which are on a non-fixed mixed C57BL/6 and 129Sv genetic background and are therefore denoted M/Mt-CK^−/−(129/Bl6)^ in this manuscript. These mice were used for breeding followed by initial echocardiography and haemodynamic studies reported in Fig. 1, with stock C57BL/6 mice used as control.

Female heterozygotes were backcrossed in our laboratory for ten generations with male C57BL/6J mice obtained from Harlan UK to produce M-CK^−/−^ and M/Mt-CK^−/−^ mice on a pure genetic background. These mice were produced by heterozygous mating, so that littermates could be used as the appropriate wild-type controls (WT). All investigations were performed when mice were approximately 1-year-old. Mice were kept under specific pathogen-free conditions in individually ventilated cages with 12-h light–dark cycle, controlled temperature (20–22 °C), and fed standard chow and water ad libitum. This investigation conforms with UK Home Office Guidance on the Operation of the Animals (Scientific Procedures) Act, 1986.

### Echocardiography

Mice were anaesthetised with 4 % isoflurane in medical oxygen and maintained on a nose-cone at 1–1.5 %. They were placed supine on a homoeothermic mat, chest fur shaved and parasternal short and long-axis images obtained. M/Mt-CK^−/−(129/Bl6)^ mice were imaged with an Agilent Sonos 5500 equipped with a 7/15 MHz linear-array transducer, while all subsequent imaging was obtained under identical conditions using a Visualsonics Vevo 2100 with 22–55 MHz transducer.

### Left ventricular haemodynamics

Approximately 1 week after echocardiography, mice were anaesthetised with 4 % isoflurane in medical oxygen and maintained on a nose-cone at 1–1.5 % on a homoeothermic mat. The left ventricle was cannulated via the right carotid artery using a 1.4-F Millar Mikro-Tip cannula (SPR-839, Millar Instruments, Houston, Texas). The right jugular vein was cannulated with stretched polyethylene tubing for infusion of dobutamine (16 ng/g body weight/min) to test contractile reserve. Measurements were obtained after 15 min of equilibration via a Powerlab 4SP data acquisition system (ADInstruments, UK). At the end of the experiment, the heart and other organs were excised, washed in heparinised saline, blotted and weighed. Left ventricular samples were snap frozen in liquid nitrogen and stored at −80 °C.

### Body composition

Body composition analysis was carried out by non-invasive magnetic resonance relaxometry in conscious restrained mice using an EchoMRI-100 system (Active Field Resources, Houston, Texas). Accumulation factor was for extra-high precision (3×) resulting in a scan time of approximately 2.5 min.

### Biochemical and molecular measurements

Total creatine and total CK, CK isoenzyme and citrate synthase activities were measured from LV homogenates as previously described [21]. The activity of total adenylate kinase (AK) was quantified spectrophotometrically as described in [1]. Total RNA was extracted from LV tissue and gene expression of LVH markers quantified using real-time RT-PCR as previously described [15].

### Data analysis and statistics

All data were analysed blind to genotype by a single experienced operator. Data are expressed as mean ± standard deviation throughout. Comparison between two groups was by student’s *t* test, and between three groups by one-way analysis of variance (ANOVA) using Bonferroni’s correction for multiple comparisons. Differences were considered significant when *P* < 0.05.

## Results

### In vivo cardiac phenotype of standard mixed background CK double knockout mouse

All previously published studies have reported on M/Mt-CK^−/−(129/Bl6)^ mice with a mixed C57BL/6 and 129Sv genetic background. When these are maintained by KO × KO breeding, there is no appropriate littermate control, and standard C57BL/6 mice have historically been used for this purpose. However, there are reported differences between C57BL/6 and 129Sv strains for blood pressure and LV mass [8], so we sought to recapitulate the finding of LV hypertrophy in M/Mt-CK^−/−(129/Bl6)^ mice under our own standard laboratory conditions and to determine whether choice of control had any influence on the results obtained. Furthermore, we performed in vivo LV haemodynamic measurements, which have never previously been reported for M/Mt-CK^−/−(129/Bl6)^ mice.

At 1+ years of age (mean 58 weeks), male M/Mt-CK^−/−(129/Bl6)^ mice had significantly higher LV weight despite lower body weight and regardless of whether C57BL/6 (+27 %; *P* < 0.001) or 129Sv (+21 %; *P* < 0.01) were used as controls (Fig. 1a). This difference was still evident after desiccation, suggesting it was not due to oedema (LV dry weight: 31 ± 2 in C57BL/6 versus 38 ± 6 mg in M/Mt-CK^−/−(129/Bl6)^; *P* = 0.008). LV hypertrophy was less pronounced in females, but remained significant (+17 % versus C57BL/6; *P* < 0.05). Echocardiography showed that male M/Mt-CK^−/−(129/Bl6)^ mice had a +16 % larger cavity cross-sectional area during diastole indicating significant LV dilatation (Fig. 1b), exhibited contractile dysfunction (d*P*/d*t*_max_ −31 %), end-diastolic pressures elevated 4.6-fold and significant pulmonary congestion compared with control mice (Fig. 1c–e), all indicative of congestive heart failure (see supplemental data table 1 for detailed analysis). In female mice, there were clear trends towards LV dysfunction for all these parameters, but differences were not statistically significant (Table 1).

**Table 1:** Morphometry and in vivo cardiac function in 1-year-old female mice on a mixed genetic background

Females	C57BL/6	M/Mt-CK^−/− (129/Bl6)^	*P*
Morphometric parameters	*n* = 6	*n* = 6	
Age (weeks)	58 ± 2	56 ± 8	0.57
Body weight (g)	30 ± 4	28 ± 4	0.55
Tibial length (mm)	18.4 ± 0.4	19.0 ± 0.4	0.02
LV weight (mg)	80 ± 6	94 ± 14	0.048
RV weight (mg)	21 ± 2	23 ± 4	0.31
Lung weight (mg)	144 ± 9	159 ± 16	0.07
Liver weight (g)	1.158 ± 0.125	1.182 ± 0.243	0.83
Kidneys (mg)	273 ± 18	368 ± 56	0.003
Haemodynamics	*n* = 6	*n* = 4	
Aortic pressure—systolic (mmHg)	96 ± 5	89 ± 6	0.09
Aortic pressure—diastolic (mmHg)	65 ± 6	58 ± 7	0.13
Aortic pressure—mean (mmHg)	79 ± 4	74 ± 6	0.14
LV end-systolic pressure (mmHg)	98 ± 5	92 ± 7	0.93
LV end-diastolic pressure (mmHg)	4.8 ± 2.9	9.3 ± 4	0.08
d*P*/d*t*_max_ (mmHg/s)	8,684 ± 1,526	6,983 ± 689	0.07
d*P*/d*t*_min_ (mmHg/s)	−8,546 ± 1,506	−6,770 ± 1,033	0.08
Heart rate (bpm)	456 ± 35	441 ± 18	0.46
Echocardiography	*n* = 6	*n* = 6	
End-diastolic area (cm^2^)	0.095 ± 0.012	0.101 ± 0.013	0.42
End-systolic area (cm^2^)	0.042 ± 0.014	0.041 ± 0.013	0.92
Fractional area change ( %)	57 ± 10	59 ± 11	0.69
Myocardial cross-sectional area (cm^2^)	0.102 ± 0.017	0.135 ± 0.011	0.002

Values are mean ± standard deviation, with *P* values for unpaired Student’s *t* test

### In vivo haemodynamics in 1-year-old CK knockout mice on a pure C57BL/6 background

All subsequent experiments were obtained in M-CK^−/−^ and M/Mt-CK^−/−^ mice that were backcrossed in our laboratory with C57BL/6 stock mice for >10 generations. Comparisons were made with a single wild-type littermate control group. Measurement of protein activity in LV homogenates confirmed loss of target genes, resulting in residual CK activity of 33 % in M-CK^−/−^ and 0.02 % in M/Mt-CK^−/−^ mice. There was an increase in BB-CK isoenzyme activity of 33 and 100 % for M-CK^−/−^ and M/Mt-CK^−/−^ hearts, respectively (Table 2), but as this represents only 2 % of normal total CK activity; however, the significance is unclear. There were no compensatory changes in either adenylate kinase or citrate synthase activities. Total LV creatine levels were indistinguishable from wild type (*P* = 0.46).

**Table 2:** Enzyme activities in left ventricular tissue from M-CK^−/−^ and M/Mt-CK^−/−^ mice on a pure C57BL/6J genetic background

Enzyme activity (IU/mg protein)	WT *n* = 11	M-CK^−/−^ *n* = 10	M/Mt-CK^−/−^ *n* = 9
Total CK	5.7 ± 0.8	1.9 ± 0.3^†^	0.12 ± 0.02^†#^
Mt-CK	2.3 ± 0.4	1.8 ± 0.3^‡^	0
MM-CK	3.1 ± 0.4	0	0
MB-CK	0.18 ± 0.06	0	0
BB-CK	0.06 ± 0.02	0.08 ± 0.01*	0.12 ± 0.02^†#^
Total adenylate kinase	2.4 ± 0.5 (12)	2.4 ± 0.1 (5)	2.2 ± 0.8 (9)
Citrate synthase	0.88 ± 0.18 (12)	0.86 ± 0.12 (12)	0.77 ± 0.20 (12)

Mean ± standard deviation

Number of samples is indicated in the headings except where included in parenthesis

* Denotes *P* < 0.05, ^‡^ *P* < 0.001 and ^†^ *P* < 0.0001 compared to wild type. ^#^ Denotes *P* < 0.001 for M-CK^−/−^ versus M/Mt-CK^−/−^

There were no significant differences between male and female mice for haemodynamic parameters when compared within genotypes, and therefore both sexes were analysed together. However, a detailed breakdown of these parameters for males and females is provided in supplemental data tables 2 and 3, respectively. Total animal numbers and sex ratios were: WT *n* = 26 (12 M/14 F), M-CK^−/−^ *n* = 31 (14 M/17 F) and M/Mt-CK^−/−^ *n* = 30 (12 M/18 F). Mean age was 55 weeks in all groups (range 52–59 weeks).

**Fig. 2:**
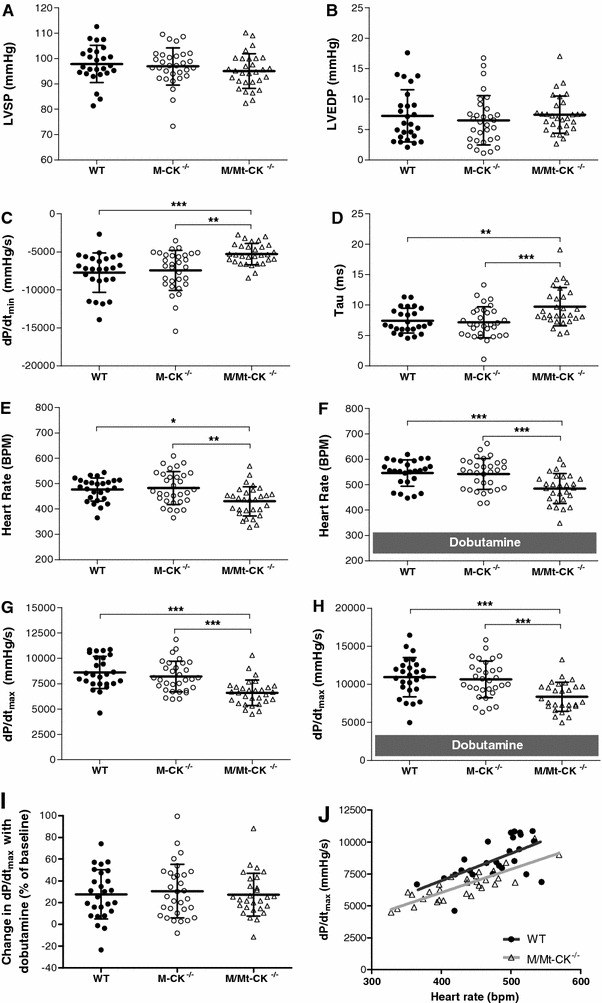
Left ventricular in vivo haemodynamics at 1 year of age in wild-type (*n* = 26), M-CK^−/−^ (*n* = 31) and M/Mt-CK^−/−^ (*n* = 30) mice on a pure C57BL/6 genetic background. **a**, **b** There were no significant differences in LV end-systolic (LVSP) and end-diastolic pressure (LVEDP), respectively; **c**, **d** diastolic function was impaired in M/Mt-CK^−/−^ hearts for both maximal rate of pressure drop (d*P*/d*t*_min_) and isovolumetric constant of relaxation (tau). **e**, **f** Heart rate was significantly lower in M/Mt-CK^−/−^ hearts, both at baseline and under maximal β-adrenergic stimulation (16 ng/g Bwt/min dobutamine). (**g**, **h**) Likewise, contractility was impaired as measured by maximal rate of pressure development (d*P*/d*t*_max_) at baseline and with dobutamine infusion, but not when measured as percentage change from baseline values (**i**). **j** There was a positive correlation between contractility (d*P*/d*t*_max_) and heart rate for both WT and M/Mt-CK^−/−^ mice (*r* = 0.61, *P* = 0.001 and *r* = 0.84, *P* < 0.0001 respectively). Linear regression analysis indicated no difference between slopes, but significantly different intercepts (*P* = 0.0004), suggesting that impaired contractility in M/Mt-CK^−/−^ mice was not secondary to lower heart rate. Mean ± SD; comparison by one-way ANOVA with Bonferroni correction for multiple comparisons; * denotes *P* < 0.05, **P < 0.01 and ***P < 0.001

Haemodynamic parameters were indistinguishable between WT and M-CK^−/−^ mice (Fig. 2). Compared to wild-type littermates, M/Mt-CK^−/−^ mice had normal LV end-systolic and end-diastolic pressures (Fig. 2a, b), but had significantly impaired indices of relaxation (d*P*/d*t*_min_ 31 % lower and tau 31 % longer than WT; Fig. 2c, d) and contraction (d*P*/d*t*_max_ 23 % lower than WT; Fig. 2g). Resting heart rate was also significantly lower than WT (10 %; Fig. 2e). Under conditions of maximal β-adrenergic stimulation using dobutamine infusion, both maximum heart rate and d*P*/d*t*_max_ increased in M/Mt-CK^−/−^ mice, but remained significantly impaired compared to WT (11 and 24 % lower, respectively; Fig. 2f, h). Thus, the relative differences in absolute values were maintained, but all groups showed similar contractile reserve as a percentage of resting baseline values, i.e. increasing d*P*/d*t*_max_ from baseline by 27 % (Fig. 2i).

The lower heart rate in M/Mt-CK^−/−^ mice may account for some (or all) of the difference observed in contractility, i.e. by occupying different positions on the force–frequency response curve. To investigate this further, d*P*/d*t*_max_ was correlated with heart rate (Fig. 2j) for both genotypes: WT Pearson *r* = 0.61, *P* = 0.001; M/Mt-CK^−/−^ Pearson *r* = 0.84, *P* < 0.0001. A runs test indicated that neither relationship deviated significantly from linearity; therefore, linear regression analysis was used to compare WT with M/Mt-CK^−/−^. Equation of line for WT was *y* = 21.3*x* – 1,562 and for M/Mt-CK^−/−^ *y* = 18.3*x* − 1,273. There was no difference in the slopes (*P* = 0.58), but the difference in elevation was highly significant (*P* = 0.0004), indicating that WT and M/Mt-CK^−/−^ hearts do not share the same relationship for d*P*/d*t*_max_ versus heart rate, but instead run in parallel (i.e. for any given heart rate, d*P*/d*t*_max_ is lower in M/Mt-CK^−/−^ hearts).

Echocardiographic examination of M/Mt-CK^−/−^ mice on the pure genetic background did not reveal any difference in cardiac volumes or in ejection fraction and cardiac output (Table 3). Pulmonary artery acceleration and ejection times were not different suggesting normal RV function and absence of pulmonary congestion. Transmitral Doppler was also indistinguishable from wild-type littermates suggesting that LV compliance was unaltered (Table 3).

**Table 3:** Echocardiography at 1 year of age in male M/Mt-CK^−/−^ mice on a pure C57BL/6J genetic background

	WT	M/Mt-CK^−/−^	*P*
Left ventricular parameters	*n* = 6 (4 M/2 F)	*n* = 9 (6 M/3 F)	
Heart rate (bpm)	493 ± 39	493 ± 46	0.98
End-diastolic volume (μl)	62 ± 14	61 ± 15	0.94
End-systolic volume (μl)	29 ± 8	27 ± 11	0.80
Stroke volume (μl)	33 ± 10	34 ± 8	0.87
Cardiac output (ml/min)	16.2 ± 4.3	16.7 ± 4.6	0.83
Ejection fraction (%)	53 ± 7	56 ± 9	0.58
Wall thickness (mm)	0.90 ± 0.05	0.93 ± 0.07	0.34
Pulmonary artery Doppler			
PAT—acceleration time (ms)	21 ± 3	21 ± 4	0.75
PET—ejection time (ms)	60 ± 8	57 ± 7	0.45
PAT/PET	0.36 ± 0.04	0.36 ± 0.04	0.87
Transmitral Doppler			
E wave (mm/s)	543 ± 168	660 ± 201	0.32
A wave (mm/s)	358 ± 143	381 ± 157	0.80
E/A	1.6 ± 0.5	1.8 ± 0.3	0.41

Values are mean ± standard deviation, with *P* values for unpaired Student’s *t* test

### Absence of LV hypertrophy on a pure C57BL/6 background

**Fig. 3:**
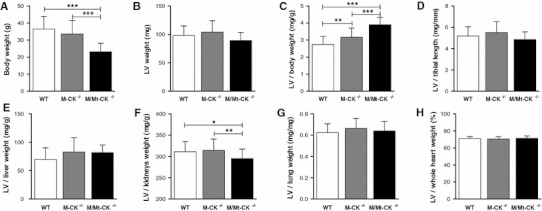
Analysis of postmortem left ventricular (LV) weight at 1 year of age in wild-type (*n* = 26), M-CK^−/−^ (*n* = 31) and M/Mt-CK^−/−^ (*n* = 30) mice on a pure C57BL/6 genetic background. (**a**) Body weight is significantly lower in M/Mt-CK^−/−^ mice, but with normal absolute LV weight (**b**), this results in a higher LV/body weight ratio (**c**). However, this difference is driven by changes in body composition affecting body weight, since LV weight is not elevated when compared with tibial length (**d**), or other major organs such as liver (**e**), kidneys (**f**) and lung (**g**) and as percentage of whole heart weight (**h**). Mean ± SD; * denotes *P* < 0.05, **P < 0.01 and ***P < 0.001

**Fig. 4:**
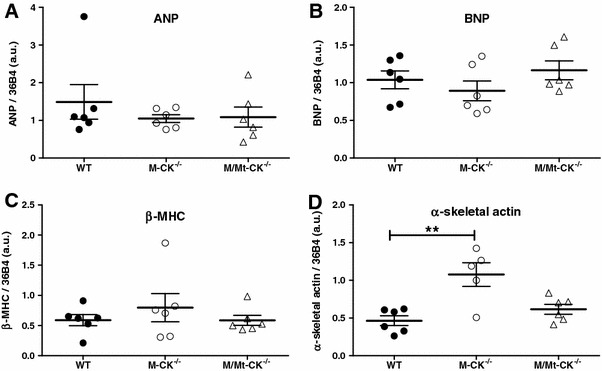
Relative gene expression of hypertrophy markers in left ventricular tissue from 1-year-old wild-type, M-CK^−/−^ and M/Mt-CK^−/−^ mice on a pure C57BL/6 genetic background (*n* = 3 male + *n* = 3 female of each genotype). *ANP* atrial natriuretic peptide, *BNP* brain natriuretic peptide, *β-MHC* β-myosin heavy chain. Mean ± SD; ** denotes *P* < 0.01

Postmortem LV weight was not elevated in either M-CK^−/−^ or M/Mt-CK^−/−^ mice, but body weight was 37 % lower in M/Mt-CK^−/−^ mice, resulting in a high LV/body weight ratio (Fig. 3a–c). However, LV weight was in proportion to tibial length and to other major organs such as liver, kidneys, lungs and total heart weight (Fig. 3d–h). The same pattern was observed in both males and females when analysed separately (see supplemental data tables 2 and 3). An absence of LVH was confirmed on a molecular level by quantitative real-time RT-PCR to measure mRNA expression of common hypertrophy markers, which were not up-regulated (Fig. 4). Surprisingly, α-skeletal actin was elevated in M-CK^−/−^ compared to WT littermate controls; however, such a change in isolation does not suggest hypertrophy.

### Body composition

**Fig. 5:**
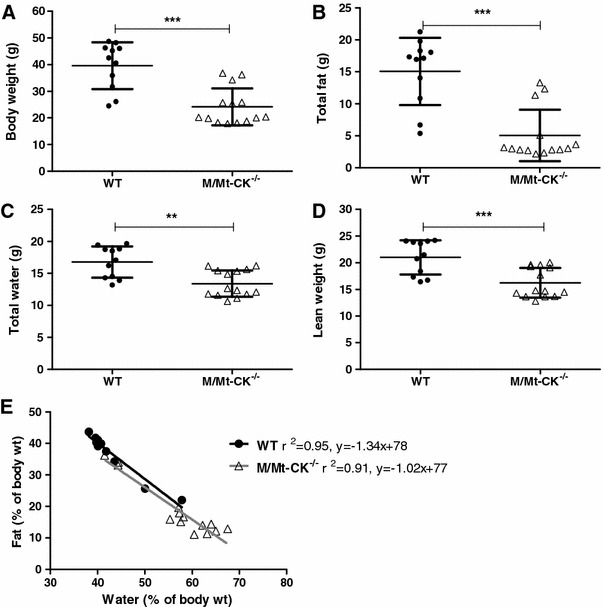
Body composition analysis by magnetic resonance relaxometry at 1-year of age in wild-type (*n* = 11) and M/Mt-CK^−/−^ (*n* = 14) mice on a pure C57BL/6 genetic background. Body weight (**a**); total body fat (**b**); total body water (**c**); lean weight (**d**). The relationship between percentage body fat and percentage body water was preserved in M/Mt-CK^−/−^ mice with a linear relationship indistinguishable from wild type. Mean ± SD; ** denotes *P* < 0.01 and ***P < 0.001

Differences in body weight were investigated further by magnetic resonance relaxometry in WT and M/Mt-CK^−/−^ mice. As suggested by organ weight analysis, differences in body weights were driven by altered body composition and in particular by drastically reduced fat in M/Mt-CK^−/−^ mice. This was accompanied to a lesser extent by lower total body water and lean weight (Fig. 5a–e). These highly significant differences were observed in both male and female mice (supplemental data table 4). There was a linear relationship between percentage body fat and body water for both WT and M/Mt-CK^−/−^ mice that was indistinguishable, suggesting that they are at different ends of the same continuum (Fig. 5e).

## Discussion

In this study, we show that the in vivo phenotype is highly dependent on genetic background, gender and age, and that there are major changes to body weight and composition. Previous reports of CK knockout mice have utilised strains on a mixed, non-fixed, genetic background, have seldom reported male-to-female ratios, and have not looked beyond 41 weeks of age. These variables are likely to explain why reports in this strain, for example relating to LV hypertrophy, have at times been contradictory.

### Male M/Mt-CK^−/−^ on a mixed genetic background develop heart failure

M/Mt-CK^−/−(129/Bl6)^ mice (i.e. on a standard mixed background) have previously been studied up to 41 weeks of age, at which point they had extensive LVH, mild dilatation and normal ejection fraction by MRI [16, 17]. We have now demonstrated that at 1 year of age this compensated hypertrophy eventually progresses to heart failure, but only in males, with females much less severely affected. Since previous studies have used stock C57BL/6 as a control for Mt-CK^−/−(129/Bl6)^ mice, we also demonstrated that the choice of control, whether C57BL/6 or 129Sv, does not affect the extent of LVH observed. Previous studies measured cardiac function using MRI, and in our experience it is not unusual to find minimal differences in global imaging parameters, yet still observe large differences in LV pressures. For example, we have previously shown that creatine-deficient mice have normal ejection fraction, but impaired pressure-generating capacity [26]. This should not be surprising, as these parameters report very different phases in the cardiac cycle.

Creatine kinase-deficient mice survive for a long time with only mild dysfunction, so obviously CK activity is not obligatory for cardiac function, but the finding that male M/Mt-CK^−/−(129/Bl6)^ mice eventually develop heart failure suggests that loss of CK places a chronic strain on the heart and that gender modulates the effects of CK depletion. Multiple changes have been described in the ageing heart that impact on its ability to tolerate stress [5], and this may result in the gradual unmasking of the functional phenotype over time. A wide range of potentially compensatory adaptations have been described in M/Mt-CK^−/−(129/Bl6)^ mice to explain the mild early phenotype, for example, closer juxtaposition of mitochondria with myofilaments, thereby reducing diffusion distance [13], coupling of glycolysis with the SERCA pump [4] and increased mitochondrial capacity with flux through alternative phosphotransfer pathways [9]. It may be that these changes are inefficient or energy costly and therefore cannot be maintained as a compensatory mechanism in the long term. This also suggests that reduced CK activity, as occurs in the failing heart [14] and in viral myocarditis [10], may indeed contribute to disease progression. Although CK dysfunction is not as severe as in the double knockout, the effect is likely to be amplified when overlaid on existing organic disease and in combination with gross changes in cellular processes, inflammation and neuro-endocrine signalling.

### M/Mt-CK^−/−^ on a pure genetic background have a milder cardiac phenotype

We have previously shown that genetic background has a major effect on the phenotype of Mt-CK^−/−^ mice [15]. We therefore backcrossed M-CK^−/−^ and M/Mt-CK^−/−^ lines until they were congenic with C57BL/6 mice (theoretically 99.9 % identical). This did not alter the phenotype in M-CK^−/−^ mice, which remained mild to non-existent even when studied at 1-year of age. However, M/Mt-CK^−/−^ mice had a less severe phenotype compared to M/Mt-CK^−/−(129/Bl6)^; most notably, they had normal end-diastolic pressure and an absence of LVH and pulmonary congestion. It is likely that this disparity purely reflects genetic differences rather than environmental or experimental influences such as diet or pathogen status, since M/Mt-CK^−/−(129/Bl6)^ obtained from Würzburg but kept in our laboratory developed LV hypertrophy as expected. Clearly, the backcrossed mice are no longer in heart failure, which suggests the existence of powerful genetic modifiers in the C57BL/6 background to account for this. Future identification of these modifiers may suggest therapeutic targets for heart failure.

A lack of LVH is in agreement with our previous findings in Mt-CK^−/−^ mice, which had up-regulation of M-CK and citrate synthase as potential compensatory pathways [15]. We predicted that backcrossed M/Mt-CK^−/−^ mice would therefore have a more pronounced phenotype, since they have no M-CK with which to compensate with. This does appear to be the case since backcrossed M/Mt-CK^−/−^ had impaired indices of isovolumetric function that were not observed in Mt-CK^−/−^ mice at the same age. A potential compensatory role for increased mitochondrial capacity (as suggested by elevated citrate synthase) was observed in our backcrossed Mt-CK^−/−^ mice [15] and also recently in the M/Mt-CK^−/−(129/Bl6)^ strain [9]. However, we did not observe an increase in citrate synthase activity in our backcrossed M/Mt-CK^−/−^ strain even though they had a milder phenotype, which argues against a compensatory role.

### Genetic background and the choice of control

It is increasingly recognised that genetic background can have a major influence on observed phenotypes in terms of both normal cardiac function [8, 31] and response to pathological stress [3], and we have previously shown this to be true for Mt-CK^−/−^ mice [15]. This underlines the importance of using appropriate controls in mouse studies. The optimal control for a double knockout mouse is to use heterozygous mating to produce double WT littermates for comparison, and this is the approach we took for the mice on a pure C57BL/6 background. This would also be optimal for mice on a non-fixed, mixed background, even though random mixing will mean that wild-type mice are not genetically identical. However, we chose to use stock C57BL/6 as controls for the experiments on M/Mt-CK^−/−(129/Bl6)^ mice, because we wanted to emulate the conditions used in previous studies to determine whether we could recapitulate their findings. The limitations of using a poor control in those studies are therefore shared by our experiments on M/Mt-CK^−/−(129/Bl6)^ mice.

### Whole body phenotype

An unexpected finding, not previously described in the literature, was lower body weight observed in M/Mt-CK^−/−^ mice regardless of genetic background (although not in female M/Mt-CK^−/−(129/Bl6)^). Using non-invasive body composition analysis, we established that this was mainly driven by reduced body fat in knockout mice. Water content was also reduced in M/Mt-CK^−/−^ mice; however, water is linearly related to fat over a wide range of body compositions in the mouse [6], therefore the preservation of the fat-to-water relationship suggests that this change is secondary to alterations in fat content. Although less pronounced, this pattern is very similar to creatine-free (GAMT knockout) mice [23] and indicates that disruption of the CK system has profound effects on whole body metabolism.

## Conclusions

Mice with chronic deficiency of both muscle- and mitochondrial-creatine kinase develop compensated LV hypertrophy that eventually progresses to congestive heart failure. The extent of this phenotype is highly dependent on genetic background, gender and age. That a primary defect in the creatine kinase system can, in itself, result in heart failure lends credence to the hypothesis that impaired CK activity observed in the failing heart may contribute to disease progression and is therefore a target for therapeutic intervention.

## Electronic supplementary material

Below is the link to the electronic supplementary material.

Supplementary material 1 (DOCX 18 kb)
