# Supplementary material for: Chronic creatine kinase deficiency eventually leads to congestive heart failure, but severity is dependent on genetic background, gender and age
Source: Basic Res Cardiol. 2012 Jul 4;107(5):276. doi: 10.1007/s00395-012-0276-2 (PMC3442167; doi:10.1007/s00395-012-0276-2)
Supplement: Supplementary file 1 — Supplementary material 1 (DOCX 18 kb) [file 395_2012_276_MOESM1_ESM.docx]

**Data Supplement**

**Supplementary Table 1** Morphometry and in vivo cardiac function in 1-year old *male* mice on a *mixed genetic background*.

| **MALES** | **C57BL/6** | **M/Mt-CK^-/- (129/Bl6)^** | ***P*** |
| --- | --- | --- | --- |
| *Morphometric parameters* | n = 7 | n = 9 |  |
| Age (weeks) | 57 ± 1 | 58 ± 7 | 0.68 |
| Body weight (g) | 42 ± 5 | 33 ± 2 | 0.0003 |
| Tibial length (mm) | 18.8 ± 0.2 | 18.9 ± 0.2 | 0.23 |
| LV weight (mg) | 122 ± 6 | 154 ± 16 | 0.0002 |
| RV weight (mg) | 32 ± 3 | 35 ± 4 | 0.07 |
| Lung weight (mg) | 173 ± 8 | 192 ± 9 | 0.0005 |
| Liver weight (g) | 2.055 ± 0.645 | 1.627 ± 0.161 | 0.07 |
| Kidneys (mg) | 457 ± 30 | 544 ± 40 | 0.0003 |
|  |  |  |  |
| *Haemodynamics* | n = 5 | n = 6 |  |
| Aortic pressure – systolic (mmHg) | 94 ± 5 | 104 ± 10 | 0.09 |
| Aortic pressure – diastolic (mmHg) | 62 ± 5 | 66 ± 12 | 0.43 |
| Aortic pressure – mean (mmHg) | 76 ± 4 | 83 ± 10 | 0.20 |
| LV end-systolic pressure (mmHg) | 100 ± 4 | 100 ± 9 | 0.93 |
| LV end-diastolic pressure (mmHg) | 4.5 ± 2.2 | 20.8 ± 13 | 0.019 |
| dP/dt_max_ (mmHg/s) | 8391 ± 1551 | 5802 ± 1471 | 0.02 |
| dP/dt_min_ (mmHg/s | -7335 ± 992 | -5005 ± 1044 | 0.004 |
| Heart rate (bpm) | 461 ± 44 | 431 ± 17 | 0.16 |
|  |  |  |  |
| *Echocardiography* | n = 7 | n = 9 |  |
| End-diastolic area (cm^2^) | 0.131 ± 0.017 | 0.151 ± 0.014 | 0.02 |
| End-systolic area (cm^2^) | 0.066 ± 0.018 | 0.083 ± 0.017 | 0.07 |
| Fractional area change (%) | 50 ± 11 | 45 ± 10 | 0.36 |
| Myocardial cross-sectional area (cm^2^) | 0.139 ± 0.012 | 0.193 ± 0.02 | 0.00002 |

Values are mean ± standard deviation, with *P* values for unpaired Student’s t-test.

**Supplementary Table 2** Morphometry and in vivo cardiac function in 1-year old male wild-type, M-CK^-/-^ and M/Mt-CK^-/-^ mice on a pure C57BL/6J genetic background.

| **MALES** | **WT** | **M-CK^-/-^** | **M/Mt-CK^-/-^** | ***P*** |
| --- | --- | --- | --- | --- |
| *Morphometric parameters* | n = 12 | n = 14 | n = 11 |  |
| Age (weeks) | 56 ± 1 | 55 ± 2 | 55 ± 2 | 0.41 |
| Body weight (g) | 42 ± 5 | 41 ± 5 | 28 ± 5**** | <0.0001 |
| Tibial length (mm) | 19.1 ± 0.2 | 19.0 ± 0.2 | 18.6 ± 0.3**** | <0.0001 |
| LV weight (mg) | 111 ± 16 | 120 ± 17 | 105 ± 8* | 0.048 |
| RV weight (mg) | 29 ± 3 | 32 ± 6 | 27 ± 3 | 0.054 |
| Lung weight (mg) | 167 ± 13 | 166 ± 14 | 145 ± 11*** | 0.0002 |
| Liver weight (g) | 1.820 ± 0.491 | 1.780 ± 0.506 | 1.433 ± 0.210 | 0.08 |
| Kidneys (mg) | 363 ± 61 | 381 ± 56 | 369 ± 47 | 0.70 |
|  |  |  |  |  |
| *Haemodynamics* | n = 12 | n = 14 | n = 11 |  |
| LV end-systolic pressure (mmHg) | 101 ± 6 | 96 ± 7 | 96 ± 5 | 0.07 |
| LV end-diastolic pressure (mmHg) | 7.6 ± 3.8 | 5.6 ± 3.9 | 7.1 ± 2.5 | 0.32 |
| dP/dt_max_ (mmHg/s) | 8665 ± 1359 | 8312 ± 1546 | 6629 ± 693** | 0.001 |
| dP/dt_min_ (mmHg/s | -7718 ± 1752 | -7832 ± 2411 | -5340 ± 1086* | 0.004 |
| Heart rate (bpm) | 563 ± 43 | 560 ± 58 | 506 ± 54* | 0.02 |
| Stimulated dP/dt_max_ (mmHg/s) | 11460 ± 2455 | 11513 ± 2096 | 8961 ± 2039* | 0.01 |
| Stimulated Heart rate (bpm) | 485 ± 45 | 490 ± 71 | 442 ± 34 | 0.07 |

Values are mean ± standard deviation, with *P* values for one-way analysis of variance. Bonferroni correction for multiple comparisons * denotes P<0.05, ** P<0.01, *** P<0.001, ****P<0.0001 compared to WT.

**Supplementary Table 3** Morphometry and in vivo cardiac function in 1-year old female wild-type, M-CK^-/-^ and M/Mt-CK^-/-^ mice on a pure C57BL/6J genetic background.

| **FEMALES** | **WT** | **M-CK^-/-^** | **M/Mt-CK^-/-^** | ***P*** |
| --- | --- | --- | --- | --- |
| *Morphometric parameters* | n = 14 | n = 17 | n = 19 |  |
| Age (weeks) | 55 ± 1 | 55 ± 1 | 55 ± 2 | 0.76 |
| Body weight (g) | 32 ± 5 | 28 ± 4 | 20 ± 2**** | <0.0001 |
| Tibial length (mm) | 18.8 ± 0.3 | 18.8 ± 0.1 | 18.1 ± 0.5**** | <0.0001 |
| LV weight (mg) | 87 ± 8 | 91 ± 11 | 80 ± 7** | 0.002 |
| RV weight (mg) | 22 ± 2 | 22 ± 3 | 21 ± 3 | 0.48 |
| Lung weight (mg) | 149 ± 11 | 149 ± 12 | 137 ± 15* | 0.014 |
| Liver weight (g) | 1.225 ± 0.267 | 1.029 ± 0.215 | 0.936 ± 0.113*** | 0.0007 |
| Kidneys (mg) | 278 ± 30 | 292 ± 33 | 267 ± 22* | 0.049 |
|  |  |  |  |  |
| *Haemodynamics* | n = 14 | n = 17 | n = 19 |  |
| LV end-systolic pressure (mmHg) | 96 ± 8 | 98 ± 8 | 95 ± 8 | 0.41 |
| LV end-diastolic pressure (mmHg) | 6.9 ± 4.8 | 7.3 ± 4.1 | 7.6 ± 3.4 | 0.89 |
| dP/dt_max_ (mmHg/s) | 8553 ± 1822 | 8107 ± 1525 | 6576 ± 1503** | 0.002 |
| dP/dt_min_ (mmHg/s | -7756 ± 3197 | -7128 ± 2817 | -5276 ± 1576* | 0.02 |
| Heart rate (bpm) | 470 ± 46 | 477 ± 61 | 424 ± 68* | 0.02 |
| Stimulated dP/dt_max_ (mmHg/s) | 10492 ± 2689 | 9913 ± 2478 | 7981 ± 1788* | 0.01 |
| Stimulated Heart rate (bpm) | 532 ± 56 | 527 ± 60 | 471 ± 60* | 0.008 |

Values are mean ± standard deviation, with *P* values for one-way analysis of variance. Bonferroni correction for multiple comparisons * denotes P<0.05, ** P<0.01, *** P<0.001, ****P<0.0001 compared to WT.

**Supplementary Table 4** Body composition in 1-year old wild-type, M-CK^-/-^ and M/Mt-CK^-/-^ mice on a pure C57BL/6J genetic background.

|  | **Males** | | **Females** | |
| --- | --- | --- | --- | --- |
|  | **WT** | **M/Mt-CK^-/-^** | **WT** | **M/Mt-CK^-/-^** |
|  | n = 6 | n = 6 | n = 5 | n = 8 |
| Body weight (g) | 46.2 ± 2.2 | 30.7 ± 5.6 * | 31.8 ± 6.7 | 19.2 ± 1.0 * |
| Total Fat (g) | 18.7 ± 1.6 | 8.0 ± 4.9 * | 10.8 ± 4.9 | 2.9 ± 0.5 * |
| Total Water (g) | 18.7 ± 0.9 | 15.6 ± 0.5 * | 14.4 ± 1.1 | 11.8 ± 0.7 * |
| Lean weight (g) | 23.6 ± 1.0 | 19.2 ± 0.8 * | 17.9 ± 1.8 | 14.0 ± 0.7 * |

Values are mean ± standard deviation. Comparisons are by Student’s t-test for WT versus M/Mt-CK^-/-^ of same sex, * denotes P<0.001.
